# Colour vision restrictions for driving: an evidence-based perspective on regulations in ASEAN countries compared to other countries

**DOI:** 10.1016/j.lansea.2023.100171

**Published:** 2023-03-03

**Authors:** Ting Fang Tan, Warapat Wongsawad, Helena Hurairah, Marie Joan Loy, Wah Wah Lwin, Nor Ain Mohd Rawi, Muhamad Sidik, Andrzej Grzybowski, Rajiv Raman, Paisan Ruamviboonsuk, Anna C.S. Tan

**Affiliations:** a Singapore National Eye Centre, Singapore General Hospital, Singapore; b Department of Ophthalmology, Mettapracharak (Wat Rai Khing) Hospital, Thailand; c Ministry of Health, Brunei; d St. Luke's Medical Center, Philippines; e Department of Ophthalmology, University of Medicine 1, Yangon Eye Hospital, Myanmar; f Ophthalmology Department, Shah Alam Hospital, Selangor, Malaysia; g Indonesian Ophthalmologists Association, Ophthalmology Department, Faculty of Medicine, Universitas Indonesia, Cipto Mangunkusomo Hospital, Jakarta, Indonesia; h Department of Ophthalmology, University of Warmia and Mazury, Institute for Research in Ophthalmology, Foundation for Ophthalmology, Poznan, Poland; i Sankara Nethralaya, Chennai, India; j Department of Ophthalmology, College of Medicine, Rangsit University, Rajavithi Hospital, Bangkok, Thailand; k Singapore National Eye Centre, Singapore Eye Research Institute, Duke-NUS, Singapore

**Keywords:** Colour vision, Colour vision impairment, Colour blindness, Driving, Requirements, Global, ASEAN

## Abstract

Colour vision deficiency is an impairment in discriminating colours. Beyond occupational opportunities, colour vision-based restrictions may limit driving, which is a daily task for many people. This review aims to compare existing colour vision requirements for obtaining a driving license in southeast Asian countries to the rest of the world. Subsequently, to review existing published literature and provide evidence-based recommendations for future guidelines for colour-deficient drivers. Color vision requirements for obtaining a driving license vary widely amongst countries. While colour-deficient drivers may face mild challenges in driving, increased awareness and developing effective compensatory strategies could enable them to drive safely. The current evidence does not support a strict exclusion of all colour-deficient individuals from driving. Instead, emphasis is needed on screening to increase awareness and insight into their disability. Future studies should consider compensatory adaptive strategies that are specific for high-risk situations such as challenging driving conditions.

## Introduction

Colour vision deficiency is a spectrum of conditions characterised by the impairment in discriminating colours, which can be congenital or acquired. Congenital forms more commonly affect males compared to females (about 8% vs. 0.4%).^1^ The acquired causes of eye diseases range from cataracts, optic nerve and retinal diseases such as age-related macular degeneration, retinitis pigmentosa, diabetic retinopathy, and glaucoma. Other rarer causes include neurological insults such as stroke or space-occupying lesions.^2^

Colour vision refers to the ability to perceive and differentiate colours, as a result of the sensory response from stimulation of cone photoreceptor pigments in the retina by different wavelengths of light within the visible spectrum. There are three classes of these cone photoreceptor pigments that have peak sensitivity in the short-wavelength (blue or S-cone), middle-wavelength (green or M-cone) and long-wavelength (red or L-cone). Colour vision deficiency is classified based on a spectrum from normal trichromatism, anomalous trichromatism, and dichromatism, to monochromatism or achromatopsia. Normal trichromats have all three classes of functional cone photopigments. Anomalous trichromats have all three cone photopigments present, but one cone photopigment has an abnormal absorption spectrum, specifically tritanomaly, deuteranomaly and protanomaly respectively for blue, green, or red photopigment abnormality. Individuals with dichromatism have a loss of one class of cone photopigment, referred to as tritanopia, deuteranopia, and protanopia respectively for the absence of blue, green, or red photopigment. Individuals with monochromatism or achromatopsia have only functional blue S-cone photopigments (ie. blue cone monochromatism) or none (ie. rod monochromatism).^3^

Colour vision tests can be quantitative or qualitative. Quantitative tests include the Farnsworth-Munsell 100 hue test and the Nagel's anomaloscope,^4^ which are both sensitive and specific. Qualitative tests include the Farnsworth 15 panel and pseudoisochromatic colour plate tests, such as the Ishihara test,^5^ the Hardy Rand and Rittler (HRR) and the Mollon Reffin test.^6^

Easy and safe driving is important for people with driving as a profession. However, drivers with colour vision deficiency may have difficulty identifying the colours of traffic signals, vehicle brake lights, and interpreting street signs.^7^ With its potential to pose safety hazards to the community at large, colour-deficient individuals may face obstacles in obtaining a driver's license. In addition, pilots who have to navigate planes and colour-coded signal controls^8^ are more impacted by colour vision deficiency. Licensing authorities in these professions may require testing of colour vision as a pre-requisite.^9^

Currently, there is no established universal colour vision standard for driving across the countries. Although, visual acuity and visual field are the most tested components to obtain a driver's license worldwide,^10^ colour vision requirements for driving show significant variation across jurisdictions. Hence, in this study, we aim to understand the existing colour vision requirements for obtaining a driver's license. Furthermore, we seek to compare these requirements within the Association of Southeast Asian Nations (ASEAN) countries, namely Brunei, Cambodia, Indonesia, Laos, Malaysia, Myanmar, Philippines, Singapore, Thailand, and Vietnam, to countries across the world. We will then review the existing evidence on the impact of colour vision deficiency on driving, to explore further recommendations.

## Methods

### Search strategy and selection criteria

A literature review was performed by searching electronic databases on PubMed, National University of Singapore (NUS) database, Google, Google Scholar, ResearchGate, and information systems journal (Management Information Systems Quarterly and European Journal of Information Systems). We included articles without restrictions on publication year, from year 1966–2021; and limited to articles with full text made available and published in English. Search terms included “colour vision”, “colour vision deficiency”, “colour vision impairment”, “colour blindness”, “dichromacy”, “driving”, “driving license”, “requirements” and “global”. The Boolean rule was applied in the literature search. Existing regulations for driving licenses were obtained from government reports, research reports, websites, and journal articles. Studies considered for review had the following characteristics (a) drivers as the target population, (b) participants with colour vision deficiency, (c) performance/implications on driving. Title and abstracts were screened for potentially relevant articles, subsequently full texts were studied, and the nresults were narratively described (Cumberland et al., 2005). Reliability of the data collected was based on the inclusion of peer-review standards for research reports and journal articles, government documents for reputable government websites, and for other articles or websites adequate referencing of published peer-reviewed literature had to be included in each article to be considered.

In addition, we found that there was a lack of literature in this area originating from southeast Asia. Hence, in August 2018, a special interest group of ophthalmologists representing the ten member countries of the Association of Southeast Asian Nations (ASEAN) documented regulations of colour vision with regard to driving in their respective countries.^11^ Among the ten members of ASEAN, Brunei was not represented during the meeting, however, requirements on driving specific to Brunei were added retrospectively. The details of the current regulations in these ASEAN countries are summarised below and compared with current global regulations and any recent updates were included in 2022.

## Results

### Existing regulations for obtaining a driving license

In most countries, colour vision deficiency does not preclude an individual from obtaining a driving license for either private or commercial vehicles (Table 1). In China, individuals with daltonism are advised not to drive but no specific colour vision tests are required to be performed to acquire a driving license. In Taiwan and for commercial licenses in Canada, there is a basic requirement to be able to distinguish red, green and amber lights similar to Singapore. Driving license regulations in the USA vary in different states, with only 13 out of the 51 states with colour vision requirements (Table 2). Of the states in the USA that have colour vision requirements, different assessment tools are used such as the Stereo Optical Optec 1000 and the Keystone view colour vision tests. The Keystone view test presents three balls, each with double digits, and requires the user to read two balls correctly to pass. The Stereo Optical Optec 1000 requires the user to identify accurately the direction that the letter ‘E’ is pointing, in five out of eight blocks. These requirements mainly applied to commercial drivers only, specifically vehicles intended to transport passengers or property. Prospective drivers from countries in South America can qualify for driving with the International Driving Permit (IDP) obtained from the American Automobile Association (AAA),^23^ which requires a valid United States driving license. Member states of the European Union have their individual driving license requirements, but the European Union regulations can potentially overrule these. Based on the European Union standards, the European Commission for driving licenses excluded colour vision requirements since its First Council Directive in 1980.^38^ Of its member states, Poland still has colour vision requirements for commercial drivers, with the requirement to correctly identify red, green and yellow colours.^16^ The United Kingdom Driver and Vehicle Licensing Agency (DVLA) did not include colour vision requirements as one of the prerequisites for obtaining a driving license.^14^ There were also no colour vision requirements for driving license applications in South Africa.^24^

**Table 1:** Global colour vision requirements for obtaining a driving license (except the USA and Southeast Asia).

Country	Colour vision requirements	Further recommendations
Australia^12^	Not specified	Ophthalmologists and optometrists should advise drivers with significant colour vision deficiency about the impact on reaction to signal lights and necessary adaptations while driving.
New Zealand^13^	Not specified	Practitioners should advise drivers on impact of colour vision on driving.
United Kingdom^14^	Group 1 (car and motorcycle) and Group 2 (bus and lorry) drivers with colour vision deficiency need not notify the DVLA	Nil
European Union^15^	Not specified	Nil
Poland^16^	Commercial drivers only Able to distinguish red, green and yellow colours	Nil
Hong Kong^17^	Not specified	Nil
People's republic of China^18^	Required but not specified	Nil
Taiwan^19^	All drivers Able to distinguish red, yellow and green	Nil
Japan^20^	Not specified	Nil
India^21^	Commercial drivers only Exclude severe colour vision deficiency, but test not specified	Suggestions for traffic lights to be made colour vision friendly as per the International Commission on Illumination
Canada^22^	Not specified	The Canada Medical Association (CMA) and Canadian Council of Motor Transport Administrators (CCMTA) recommend that drivers are made aware of their colour deficiencies by their doctors.
South America^23^	Not specified	Drivers with the International Driving Permit (IDP) obtained from the American Automobile Association (AAA) allowed to drive
South Africa^24^	Not specified	Nil
Kenya^25^	Not specified	Nil

**Table 2:** Colour vision requirements for obtaining a driving license in selected states of the USA.

Country	Colour vision requirements	Further recommendations
Alabama^26^	New and commercial drivers Using Keystone view colour vision test to distinguish red, amber and green	Nil
Arizona^27^	Commercial drivers only Using Keystone view colour vision test	Nil
Maryland^28^	Commercial drivers only Using Stereo Optical Optec 1000 colour vision test	Nil
Indiana^29^	Commercial drivers only Test not specified	Nil
Montana^30^	Commercial drivers only Using Stereo Optical Optec 1000 colour vision test	Nil
Nebraska^31^	Commercial drivers only Test not specified	Nil
Ohio^32^	Required but not specified	Nil
Tennessee^33^	Commercial drivers only Using Stereo Optical colour vision test	Nil
Texas^34^	New drivers Test not specified	Nil
Massachusetts^35^	All drivers Using Stereo Optical Optec 1000 colour vision test to distinguish red, amber and green	Nil
Washington^36^	New and commercial drivers Test not specified	Nil
Wisconsin^37^	Commercial drivers only Using Stereo Optical colour vision test	Nil

In general, these colour vision regulations in the ASEAN countries are stricter, compared to the rest of the world (Table 1). Among the ten ASEAN countries represented (as shown in Table 3)^11^, eight of ten countries deny driving licenses to individuals with any colour vision impairment. Four of these countries used the Ishihara test plates for colour vision assessment (Cambodia, Malaysia, Myanmar, Indonesia), while the other four countries use three colour discrimination tests (Singapore, Thailand, Laos, Brunei). On the contrary, two other countries (Vietnam and the Philippines) had no restrictions on colour vision for driving.

**Table 3:** Colour vision requirements for obtaining a driving license in ASEAN countries.^11^

Country	Colour vision test	Authorized persons licensed to conduct testing	Exclusion criteria for driving
Brunei	Three colours discrimination test	Ophthalmologist	Unable to discriminate red, green and amber from 23 m
Cambodia	Ishihara	Ophthalmologist and ophthalmic health personnel	Any colour vision impairment^a^
Indonesia	Ishihara	General practitioners	Any colour vision impairment^a^
Malaysia	Ishihara	Land Transportation Authority and Ophthalmologist	Any colour vision impairment^a^ for commercial drivers
Myanmar	Ishihara	Land Transportation Authority	Any colour vision impairement^a^ for commercial drivers
Laos	Three colours discrimination test	Land Transportation Authority	Any colour vision impairment^a^
Philippines	None specified	N/A	None specified
Singapore	Three colours discrimination test	Land Transportation Authority and Ophthalmologists	Unable to discriminate red, green and amber colours when examined by an Ophthalmologist
Thailand	Three colours discrimination test	Land Transportation Authority	Any colour vision impairment^a^ detected by Ophthalmologist
Vietnam	None specified	N/A	None specified

a No specification of minimum scores on colour vision tests.

Over the years, there has been a notable trend towards relaxing colour vision regulations in driving. The national standard for colour vision in commercial license was introduced in Australia in 1994, later amended to exclude only protans—who have reduced sensitivity to red light^7^— and subsequently completely abandoned in 2003 due to protests.^39^ The European Commission for driving licenses excluded colour vision requirements since its First Council Directive in 1980.^38^ More recently, the Ministry of Road Transport and Highways in India amended the Central Motor Vehicle Rules in June 2020,^20^ limiting restrictions to only commercial drivers with severe deficiency, based on recommendations by All Institute India of Medical Sciences (AIIMS), New Delhi.^40^

### Evidence-based literature review on the effect of colour vision deficiency on driving

International evidence-based guidelines do not call for tighter regulations.^41^^,^^42^ A recent review by the Federal Motor Carrier Safety Administration (FMCSA) recommended no further stringent requirements beyond the colour perception of standard colours (red, green, amber).^43^ The International Council of Ophthalmology also concluded at the 2006 Congress that colour vision is not incompatible with safe driving, as the problem with recognizing traffic lights is overcome by factors such as standardized positioning or appropriately chosen colours of traffic lights.^44^ The European Commission Eyesight Working Group 2005 report pushed for the inclusion of driving requirements for twilight vision (vision in dim light conditions) and absence of double vision but did not find significant evidence to justify the inclusion of colour vision requirements.^45^

On the other hand, some previous studies have also shown that colour deficient drivers have self-reported difficulties on the road such as distinguishing traffic signals, confusing traffic lights with streetlights, and detecting brake lights.^46^^,^^47^ A significant reduction in visual range for red,^48^ may cause protans to have a reduced capacity to see red stoplights or rear lights, especially under conditions such as dim lighting or wearing sunglasses. This can be further exacerbated by slippery roads that require increased stopping distance.^49^ Moreover, as reaction time is associated with the apparent brightness of the target, light being perceptually dimmer to protans may translate to longer reaction times.^50^

However, these reported driving deficits in colour deficient drivers do not translate into higher risks for crashing compared to drivers with normal colour vision.^47^^,^^51^^,^^52^ This may be attributed to adaptive compensatory mechanisms. For example, standardised positioning of the traffic lights with more appropriately chosen colours^53^ (e.g. more bluish ‘green’ traffic lights, higher intensity red lights), may help colour deficient individuals overcome difficulties in distinguishing specific colours.^54^ Some research shows that people with colour vision disorders may self-regulate, with a preference for daytime driving and driving less regularly.^55^ Particularly in protans, a reduced capacity to detect a red target may result in a lower expectation of danger and less ability to compensate.^56^ Hence, improving awareness of their condition and the associated limitations will reduce the potential risks by encouraging the adoption of safer strategies during driving.^22^

## Discussion

### Need for more awareness and advice

Colour vision deficiency encompasses a spectrum of deficits that can have a variable impact on function.^57^ In general, most colour-deficient individuals can adapt and drive safely if they have normal visual acuity and visual fields.^52^ Hence, there has been an increasing trend globally about not excluding colour-deficient individuals from obtaining their driving license. The focus should be on improving awareness and insight into their condition and associated limitations.^56^ Colour-deficient drivers can then cultivate self-regulation, adaptive strategies and adopt safer driving practices (e.g. maintaining longer following distances, slowing down at intersections).^22^^,^^58^^,^^59^ In specific cases, such as individuals with protanopia with reduced sensitivity to the colour red and its associated reduced reaction times to brake lights,^50^ more stringent restrictions may be considered on a case-by-case basis to exclude protans from obtaining commercial driving licenses. Overall, there is limited published evidence on specific advice for colour-deficient individuals to adopt in the field of driving.

### Need for standardization of colour vision tests

Furthermore, there is no clear evidence to suggest which standardized colour vision test should be used. The anomaloscope discriminates the type and severity of colour vision deficiency, based on the patient's ability to match colours using a mixture of red and green lights based on the Rayleigh equation.^4^ It has been regarded as the gold standard, and widely used as the standard of comparison to other colour vision tests.^60^, ^61^, ^62^ One study, which used the Nagel anomaloscope, demonstrated 100% sensitivity and specificity.^63^ However, the device is not readily available in all ASEAN countries,^11^ and is expensive and technically difficult to operate.

Ishihara test is the most used screening tool for colour vision assessment in ASEAN countries, which is widely available and inexpensive with a positive predictive value of 83%.^5^ However, it has a poor correlation between the number of ‘failed’ test plates and the severity of red-green colour vision deficiency, as not all test plates have equal difficulty.^3^^,^^64^ Furthermore, Barbur et al. compared the different editions within the Ishihara test plates (ie. 38, 24, 14-plates edition), where using two or less errors to pass as per industrial routine protocols, majority of all normal trichromats passed, but 6.7% deutans and 1.6% protans that have severe colour vision deficiency can also pass for the 14 and 24-plates edition.^3^ Other common screening tests for colour vision include the Hardy Rand and Rittler 4th edition (HRR; Richmond Optical, CA), the City University test (Keeler USA, Malvern, PA) and the Mollon Reffin test (PA Vision, Margate, Kent, UK). A study by Davison and colleagues compared these tests to the anomaloscope and found that the Ishihara test performed the poorest in differentiating protanopes and deuteranopes, while the HRR and Mollon Reffin test were the most effective in identifying protanopia accurately.^6^

Other newer methods of testing have also been explored, such as a web-based test in German language using pseudoisochromatic colour plates.^65^ However, its accuracy was limited by difficulty with visual calibration to ensure exact colour reproduction. Smartphone-based applications like Eye2Phone and colour vision test applications seek to improve convenience, but once again reduced sensitivity limits its usefulness in clinical practice.^66^ These digital tests still require further validation and are not ready to replace conventional colour vision tests; however, their increased accessibility may prompt affected individuals to seek formal vision testing.

There have been existing efforts in an attempt to minimize unfair discrimination against those with deficient colour vision that can achieve levels of performance comparable to normal counterparts. The United Kingdom Civil Aviation Authority employed the Colour Assessment and Diagnosis (CAD) test to establish new minimum colour vision requirements for professional aviation pilots.^67^ The CAD test uses moving colour signals to accurately measure red-green and yellow-blue threshold sensitivities, assess the class with high specificity and estimate the severity of colour vision deficiency. Also, CAD test also checks whether the colour deficiency qualifies the minimum requirements to perform the most safety-critical task. Threshold sensitivities are measured as the number of standard normal CAD units from trichromatic normal counterparts. Use of the CAD test demonstrated that 36% deutans and 30% protans would then be included as safe to fly,^68^ who would have otherwise been excluded as per conventional colour vision tests. Similarly, the CAD test was implemented by the Transport for London since 2011 to assess underground train captains, which allowed a third of applicants who were previously rejected for failing this Ishihara test, to now be deemed safe.^69^

Barbur and colleagues also proposed a new system of colour vision categories,^3^ to quantify the severity and to identify what can be considered safe or functional based on the requirements of specific colour vision tasks in visually demanding occupations. Categories were divided based on accurate measurement of red-green and yellow-blue colour thresholds, into ‘Supernormal’ trichromatic colour vision (CV0), ‘Normal’ trichromatic colour vision (CV1), ‘Functionally normal’ trichromatic colour vision (CV2), ‘Safe’ trichromatic colour vision (CV3), ‘Poor’ red-green colour vision (CV4) and ‘Severe red-green colour deficiency (CV5); as well as for yellow-blue colour vision into ‘Supernormal’, ‘Normal’ and ‘Acquired colour deficiency’. With this system, about 22% of deutans and 1% of protans are classified as ‘Safe’ or ‘Functionally normal’. These efforts demonstrate the importance to relook at existing requirements and finetune requirements specific to different severities of colour vision deficiency. Furthermore, this prompts future studies to investigate the validity and feasibility to potentially implement these grading systems in the field of driving.

### Newer innovations to potentially narrow the gap

Moving forward, newer innovations with the advancement in technology give hope to potentially minimise the difficulties associated with abnormal colour vision. In a study in Japan, brighter blue LED lights were incorporated in an ‘X’ through red traffic lights,^70^ to which red-green colour deficient drivers can better distinguish against the contrasting ‘red’ background that they perceive as yellow, and the mark is clear even from a distance. Interestingly this blue ‘X’ cannot be seen by counterparts with normal colour vision, because colour deficient individuals have relatively higher luminous efficiency in the shorter range (ie. blue) than in the longer range (ie. red) of wavelengths.^70^ Furthermore, as deficiencies in colour vision are attributed to gene mutations coding for different components of retinal cones, there is the possibility of gene therapy as a treatment modality in the future.^71^ Existing experimental trials have demonstrated improvement in electroretinogram-investigated cone cell functionality and visually elicited behaviour.^71^

Overall, the current evidence does not support excluding colour-deficient individuals from obtaining a driving license. However, while many studies have emphasised the need for screening to increase awareness, there currently lacks evidence-based, deficiency-specific advice for colour-deficient individuals who may potentially experience difficulties. Moreover, it may be of value to further study the different demands of colour vision in specific scenarios such as commercial driving, unique driving conditions like slippery roads while raining or dim lighting at night. This can potentially guide fine-tuning of requirements and formulate specific advice to affected individuals. Hence, this suggests, that more research is required in these areas to derive updated standardised guidelines and tangible evidence-based advice for driving licensing authorities.

## Contributors

ACST formulated and led the direction of the article. WW, HH, MJL, WWL, NAMR, MS, PR, ACST contributed to the data collection and manuscript. TFT conducted the literature search and data extraction. The manuscript was revised and finalised by TFT, AG, PR, RR and ACST.

## Declaration of interests

MJL is a speaker at the 10.13039/100007819Allergan, 10.13039/100007489Bausch and Lomb, 10.13039/100004326Bayer, 10.13039/100004336Novartis, 10.13039/501100004286Santen; and investigator at 10.13039/100007819Allergan, 10.13039/100004336Novartis, Chengdu Kanghong. ACST is a consultant and speaker for 10.13039/100004326Bayer, 10.13039/100004336Novartis 10.13039/100007819Allergan, Nidek and Zeiss. She receives grant funding from Zeiss, 10.13039/100004336Novartis and Nidek. All other authors declare no other conflict of interests. The authors received no funding for this article.
